# FGF1 Fusions with the Fc Fragment of IgG1 for the Assembly of GFPpolygons-Mediated Multivalent Complexes Recognizing FGFRs

**DOI:** 10.3390/biom11081088

**Published:** 2021-07-23

**Authors:** Marta Poźniak, Weronika Zarzycka, Natalia Porębska, Agata Knapik, Paulina Marczakiewicz-Perera, Malgorzata Zakrzewska, Jacek Otlewski, Łukasz Opaliński

**Affiliations:** Department of Protein Engineering, Faculty of Biotechnology, University of Wrocław, 50-387 Wrocław, Poland; marta.latko2@uwr.edu.pl (M.P.); 281169@uwr.edu.pl (W.Z.); natalia.porebska2@uwr.edu.pl (N.P.); agata.knapik@gmail.com (A.K.); paulina.marczakiewicz@gmail.com (P.M.-P.); malgorzata.zakrzewska@uwr.edu.pl (M.Z.); jacek.otlewski@uwr.edu.pl (J.O.)

**Keywords:** FGF1, FGFR, spatial distribution, endocytosis, signaling

## Abstract

FGFRs are cell surface receptors that, when activated by specific FGFs ligands, transmit signals through the plasma membrane, regulating key cellular processes such as differentiation, division, motility, metabolism and death. We have recently shown that the modulation of the spatial distribution of FGFR1 at the cell surface constitutes an additional mechanism for fine-tuning cellular signaling. Depending on the multivalent, engineered ligand used, the clustering of FGFR1 into diverse supramolecular complexes enhances the efficiency and modifies the mechanism of receptor endocytosis, alters FGFR1 lifetime and modifies receptor signaling, ultimately determining cell fate. Here, we present a novel approach to generate multivalent FGFR1 ligands. We functionalized FGF1 for controlled oligomerization by developing N- and C-terminal fusions of FGF1 with the Fc fragment of human IgG1 (FGF1-Fc and Fc-FGF1). As oligomerization scaffolds, we employed GFPpolygons, engineered GFP variants capable of well-ordered multivalent display, fused to protein G to ensure binding of Fc fragment. The presented strategy allows efficient assembly of oligomeric FGFR1 ligands with up to twelve receptor binding sites. We show that multivalent FGFR1 ligands are biologically active and trigger receptor clustering on the cell surface. Importantly, the approach described in this study can be easily adapted to oligomerize alternative growth factors to control the activity of other cell surface receptors.

## 1. Introduction

Fibroblast growth factor receptors (FGFRs) constitute a family of four receptor tyrosine kinases (RTKs) that, together with their cognate ligands, fibroblast growth factors (FGFs), are involved in the transmission of signals from the extracellular environment to the cell interior. FGFRs are composed of a large, highly glycosylated extracellular region including acidic box (AB) and three immunoglobulin-like domains D1, D2 and D3, of which D2 and D3 are involved in FGF binding, whereas AB and D1 fulfill regulatory functions. FGFRs are anchored to the plasma membrane by a single transmembrane helix that participates in receptor dimerization. The intracellular part of FGFRs comprises a regulatory juxtamembrane region and a tyrosine kinase (TK) domain directly involved in the phosphorylation cascade [1,2]. In the prevailing model, inactive FGFRs are predominantly monomeric and dimerize upon FGF binding, resulting in large conformational changes of the receptors, leading to the transphosphorylation of key tyrosine residues within TK. These modifications provide binding sites for several proteins involved in cellular signaling, initiating the propagation of the signals through phospholipase C gamma (PLCγ), signal transducer and activator of transcription (STAT), phosphoinositide 3-kinase (PI3K)/protein kinase B (AKT)/mammalian target of rapamycin (mTOR), and Ras–Raf–MEK–ERK. The FGFR-dependent signaling controls pivotal cellular processes, such as division, motility, metabolism and death [3,4,5,6,7].

Unbalanced FGF/FGFR signaling is responsible for several developmental diseases and is a hallmark of numerous tumors [4,8,9]. Therefore, cells tightly balance FGF/FGFR units through several regulatory mechanisms that fine-tune the amplitude and duration of signals. For example, the stability of FGF/FGFR complexes determines downstream signaling and the induced cellular responses [10]. FGF/FGFR activity is modulated by a number of extracellular and plasma membrane-embedded proteins, some of which act as essential co-receptors [11,12,13,14]. Furthermore, several feedback loops participate in the downregulation of FGF/FGFR signaling [15,16]. FGFR endocytosis, which directs receptors to lysosomes for degradation, further shapes the kinetics and specificity of the signals transmitted [6,16,17].

The modulation of the spatial distribution of FGFR in the plasma membrane has recently emerged as an additional mechanism for shaping FGFR activity and cell fate. Firstly, the specificity of FGFR signaling is adjusted by the oligomeric state of the receptor. Several reports indicated that FGFRs form oligomers in the absence of ligands and these complexes are partially signaling active [18]. Secondly, the duration of FGFR-dependent signals depends on the residence time of the receptor on the cell surface. Shifting FGFR1 from monomer to dimer with a bivalent engineered antibody stimulates clathrin-mediated endocytosis and lysosomal degradation of FGFR1, shortening the receptor’s exposure at the surface [19,20]. High-order oligomerization of FGFR1 with a tetravalent engineered antibody or with FGF1 oligomers further enhances the efficiency of receptor endocytosis and accelerates its lysosomal targeting [21]. Importantly, FGFR1 clustering alters the mechanisms of receptor internalization by activating clathrin-independent, dynamin-dependent endocytic routes [21]. Thirdly, tetravalent and pentavalent FGF1 oligomers alter FGFR1 activity by promoting mitogenic signaling and inhibiting cell metabolic activity [22].

All above-mentioned effects were largely dependent on the architecture of the oligomeric ligand applied to crosslink FGFR1. Thus, it is highly likely that yet unexplored FGFR1 activities and receptor-dependent cellular effects can be triggered by novel multivalent FGFR1 ligands. Here, we report a novel strategy to generate oligomeric FGFR1 ligands by employing bivalent fusions of FGF1 with the Fc fragment of human IgG1 and GFPpolygons-protein G as oligomerization scaffolds.

## 2. Materials and Methods

### 2.1. Antibodies and Reagents

The primary antibodies directed against ERK1/2 (#9102) and phospho-ERK1/2 (pERK1/2; #9101) were from Cell Signaling (Danvers, MA, USA). Anti-tubulin primary antibody (#T6557) was from Sigma-Aldrich (St. Louis, MO, USA), anti-FGF1 primary antibody (sc-55520) was from Santa Cruz Biotechnology (Dallas, TX, USA) and anti-human IgG (Fc) antibody coupled to HRP (#4-10-20) was from KPL (Gaithersburg, MA, USA). Secondary antibodies coupled to HRP were from Jackson Immuno-Research Laboratories (Cambridge, UK).

### 2.2. Cells

The human osteosarcoma cell line (U2OS) was obtained from American Type Culture Collection (ATCC), and U2OS, stably expressing FGFR1 (U2OS-R1), were obtained as described previously [23]. Cells were cultured in 5% CO_2_ atmosphere at 37 °C in Dulbecco’s Modified Eagle’s Medium (Biowest, Nuaille, France) supplemented with 10% fetal bovine serum (Thermo Fisher Scientific, Waltham, MA, USA), antibiotics mix (100 U/mL penicillin and 100 μg/mL streptomycin) (Thermo Fisher Scientific), for U2OS-R1, additionally supplemented with 1 mg/mL geneticin (Thermo Fisher Scientific). Murine embryonic fibroblasts (NIH3T3) were from ATCC and were cultured in Dulbecco’s Modified Eagle’s Medium (Gibco) supplemented with 2% bovine serum (Thermo Fisher Scientific) and antibiotics mix (100 U/mL penicillin and 100 μg/mL streptomycin). Cells were grown in 5% CO_2_ atmosphere at 37 °C.

### 2.3. Recombinant Proteins

Plasmid pET28a_HisTag-GFPpoly_protG was a kind gift from the Jung lab, Department of Chemistry, National University in Daejeon, South Korea. The GFPpoly-ProtG (GFPpG) oligomers were expressed in *E. coli* BL21(DE3)-RIL strain (Agilent Technologies, Santa Clara, CA, USA). Cells were grown at 37 °C until OD_600_ = 0.8, then protein expression was induced by addition of 1 mM IPTG, followed by incubation of cells at 16 °C for 16 h. GFPpG oligomers were purified using nickel-charged resin, eluted with 50 mM Tris, 150 mM NaCl, 250 mM imidazole, pH 8.0. Next, GFPpG variants were separated by Blue Native-PAGE (BN-PAGE). Mixture of proteins were applied in maximum volume into gradient gel (4–13%). After the electrophoretic separation the bands representing each of GFPpG oligomers were cut out under UV light and transferred to buffer containing 20 mM Tris, 150 mM glycine, 0.02% SDS, pH 8.3 with overnight shaking at 4 °C. Then, separated GFPpG variants were dialyzed into PBS buffer. The identity and the purity of the proteins were confirmed by SDS-PAGE and Western blotting. Pure GFPpG oligomers were stored with the addition of 5% glycerol at −20 °C.

Genetic constructs for expression of Fc-FGF1 and FGF1-Fc were prepared using restriction free cloning technique. In the first part of this method the FGF1 sequence was amplified in a regular polymerase chain reaction (PCR). Primers were obtained from Sigma Aldrich and their sequences were sequentially: 5′ GAGCCTCTCCCTGTCTCCG GGTAAAATGGCTAATTACAAGAAGCCCAAAC 3′ (forward) and 5′ GAACCACTGAA CAAATGGCACTAGGTTAATCAGAAGAGACTGGCAGGGGG 3′ (reverse) for the Fc-FGF1, and 5′ GTCAGTAACGACTGGTGTCCACTCCGCTAATTACAAGAAGCCCAAACTCC 3′ (forward) and 5′ ACGGTGGGCATGTGTGAGTTTTGTCATCAGAAGAGACTGGCAGGG GGAGA 3′ (reverse) for the FGF1-Fc. The pET3c_FGF1 construct was used as a template. Amplification reactions were performed in a total volume of 50 µL. Obtained PCR product was used as a megaprimer in a second amplification reaction, introducing FGF1 in front or behind the Fc fragment coding sequence in pLEV113 plasmid. Amplification reactions were also performed in a total volume of 50 µL. Both PCR reactions were performed with use of Phusion Flash High-Fidelity PCR Mater Mix (Thermo Fisher Scientific). Obtained products were subjected to DpnI digestion (Thermo Fisher Scientific), and the correctness of genetic constructs was confirmed by DNA sequencing. The FGF1-Fc-FGF1 genetic construct was prepared via gene synthesis combined with standard molecular biology methods. Recombinant proteins were expressed in CHO-S cells. Cells were grown in serum-free PowerCHO-2CD medium (Lonza, Basel, Switzerland) supplemented with 8 mM L-glutamine (Biowest, Riverside, MO, USA) and antibiotic mix (100 U/mL penicillin and 100 µg/mL streptomycin) (Thermo Fisher Scientific). One day prior to transfection, CHO-S cells were seeded at 1.8 × 10^6^ cells/mL. On the transfection day, CHO-S cells were centrifuged and cell pellet was re-suspended in protein- and serum-free ProCHO4 medium. Plasmids encoding Fc-FGF1 or FGF1-Fc or FGF1-Fc-FGF1 (1.25 μg DNA per 1 × 10^6^ cells) and PEI (5 μg per 1 × 10^6^ cells) were diluted separately in 150 mM NaCl, mixed, and incubated at RT for 10 min prior addition to cells. Cells were incubated under standard conditions for 4 h (37 °C, 110 rpm, 8% CO_2_). After this time, the cell culture was diluted with an equal volume of PowerCHO-2CD supplemented with 4 mM L-glutamine and antibiotic mix (200 U/mL penicillin and 200 µg/mL streptomycin) to obtain the cell density of about 1 × 10^6^ cells/mL, and incubated at 32 °C. The next day, the cell culture was supplemented with 8 mM L-glutamine and finally harvested at day 7. Fc-FGF1, FGF1-Fc and FGF1-Fc-FGF1 were purified from culture supernatants using the HiTrap MabSelect SuRe column (GE Healthcare, Chicago, IL, USA). Both proteins were eluted with 0.01 M sodium citrate, pH 3.5, and neutralized with 1 M Tris-HCl, pH 9.0. Then, proteins were dialyzed into PBS buffer using the Desalting HiTrap column (GE Healthcare). The identity and the purity of the proteins were confirmed by SDS-PAGE and Western blotting.

### 2.4. Activation of FGFR1 and Receptor-Downstream Signaling Cascades

To analyze the impact of the recombinant proteins on FGFR1 activation and initiation of receptor-downstream signaling cascades, serum starved NIH3T3 cells were incubated for 15 min at 37 °C with FGF1 (100 ng/mL), as control, Fc-FGF1 (50, 100 ng/mL) or FGF1-Fc (50, 100 ng/mL) in the presence of heparin (10 U/mL). Cells were lysed in Laemmli buffer and subjected to SDS-PAGE and Western blotting.

An analogous experiment was performed for the FGF1 and GFPpG multivalent complexes. Serum starved NIH3T3 cells were incubated for 15 min at 37 °C with Fc-FGF1 or FGF1-Fc (both at 100 ng/mL) and GFPpG variants (at equimolar concentrations to the Fc) in the presence of heparin (10 U/mL). Cells were lysed in Laemmli buffer and subjected to SDS-PAGE and Western blotting.

### 2.5. Fluorescence Microscopy

For the analysis of Fc-FGF1 and FGF1-Fc cellular uptake, early endosomes in U2OS (control) and U2OS-R1 cells were labeled with Rab5a-RFP (CellLight Early Endosomes-RFP, Thermo Fisher Scientific). Next, serum-starved cells were incubated with each recombinant protein (both at 500 ng/mL) in the presence of heparin (10 U/mL) at 37 °C, 5% CO_2_ for 30 min. After this time, the internalization was stopped by cooling down cells on ice. Next, cells were fixed with 4% paraformaldehyde and permeabilized with 0.1% Triton in PBS. Zenon AF-488 (Thermo Fisher Scientific) was used for labeling of Fc-FGF1 and FGF1-Fc and NucBlue Live (Thermo Fisher Scientific) was used to fluorescently mark nuclei. The Zenon AF-488 signal was visualized with a 450/490 nm bandpass excitation filter and a 500/550 nm bandpass emission filter. The cell light early endosomes-RFP signal was visualized with a 540/552 nm bandpass excitation filter and a 575/640 nm bandpass emission filter. NucBlue Live signal was visualized with 335/383 nm bandpass excitation filter and 420/470 nm emission filter.

For the analysis of FGF1 and GFPpG multivalent complexes cellular uptake serum starved U2OS-R1 cells were incubated with each of GFPpG variant (5 µg/mL) and Fc-FGF1 or FGF1-Fc (at equimolar concentrations to the protein G in GFPpG) in the presence of heparin (10 U/mL). After 20 min of incubation on ice, the cells were moved to 37 °C, 5% CO_2_ for 45 min to allow for internalization. After this time, the internalization was stopped by cooling down cells on ice. Next, cells were fixed with 4% paraformaldehyde. DyLight 550 NHS Ester (Thermo Fisher Scientific) was used for the labeling of Fc-FGF1 and FGF1-Fc and NucBlue Live (Thermo Fisher Scientific) was used to fluorescently mark nuclei. GFP signal was visualized with a 450/490 nm bandpass excitation filter and a 500/550 nm bandpass emission filter. DyLight 550 signal was visualized with a 540/552 nm bandpass excitation filter and a 575/640 nm bandpass emission filter. NucBlue Live signal was visualized with 335/383 nm bandpass excitation filter and 420/470 nm emission filter. Wide-field fluorescence microscopy was carried out using Zeiss Axio Observer Z1 fluorescence microscope (Zeiss, Oberkochen, Germany). Images were taken using LD-Plan-Neofluar 40 × /0.6 Korr M27 objective and Axiocam 503 camera. Images were processed with Zeiss ZEN 2.3 software (Zeiss, Oberkochen, Germany) and Adobe Photoshop (Adobe, San Jose, CA, USA).

### 2.6. Flow Cytometry

U2OS-R1 cells were seeded onto 12-well plates (1 × 10^5^ cells per well) in full medium and left to attach overnight. Then, medium was removed, cells were washed with PBS buffer and starved with serum-free medium for 4 h. Next, plates were cooled on ice. Fluorescent GFPpG variants (500 ng/mL) alone or combined with Fc-FGF1 or FGF1-Fc (at equimolar concentrations to the protein G in GFPpG) were added to the cells in the presence of heparin (10 U/mL), in a serum-free medium supplemented with 1% BSA. After 20 min of incubation on ice, the cells were moved to 37 °C for 30 min to allow for internalization. Then, the medium was removed and the cells were washed with serum-free medium supplemented with 0.2% BSA pH 3.5 (three times, 5 min) and then with PBS buffer (three times, 1 min). Cells were subsequently detached with 10 mM EDTA in PBS buffer, pH 8.0, harvested by centrifugation and resuspended in PBS supplemented with 1% BSA. Cells were analyzed using a Agilent Technologies, Santa Clara, CA, USA) and NovoExpress software (ACEA Biosciences, San Diego, CA, USA).

### 2.7. Blue Native-PAGE

The formation of Fc-FGF1 or FGF1-Fc complexes with GFPpG was analyzed with Blue Native PAGE (BN-PAGE). The experiments were performed with Fc-FGF1 (5 µg), FGF1-Fc (5 µg) and their mixtures with various oligomeric forms of GFPpG (from dimer to pentamer) (5 µg) incubated for 10 min at RT. Proteins were separated using 4–13% BN-PAGE gradient gels and imaged under UV light.

### 2.8. BLI Measurements

Binding analyses of FGF1 variants (Fc-FGF1 and FGF1-Fc) to GFPpG oligomers (from dimer to pentamer) was performed using bio-layer interferometry (BLI) with ForteBio Octet K2 (Pall ForteBio, San Jose, CA, USA). The Fc-FGF1 (10 µg/mL) or FGF1-Fc (10 μg/mL) was chemically immobilized on Amine Reactive Second-Generation (AR2G) biosensors (Pall ForteBio, San Jose, CA, USA). The measurements were conducted at 25 °C for each GFPpG variant (20 μg/mL) in PBS buffer supplemented with 0.2% BSA and 0.05% Triton X-100. Association and dissociation phases were monitored by 300 s.

## 3. Results

### 3.1. Strategy for Controlled Oligomerization of FGF1

**Figure 1:**
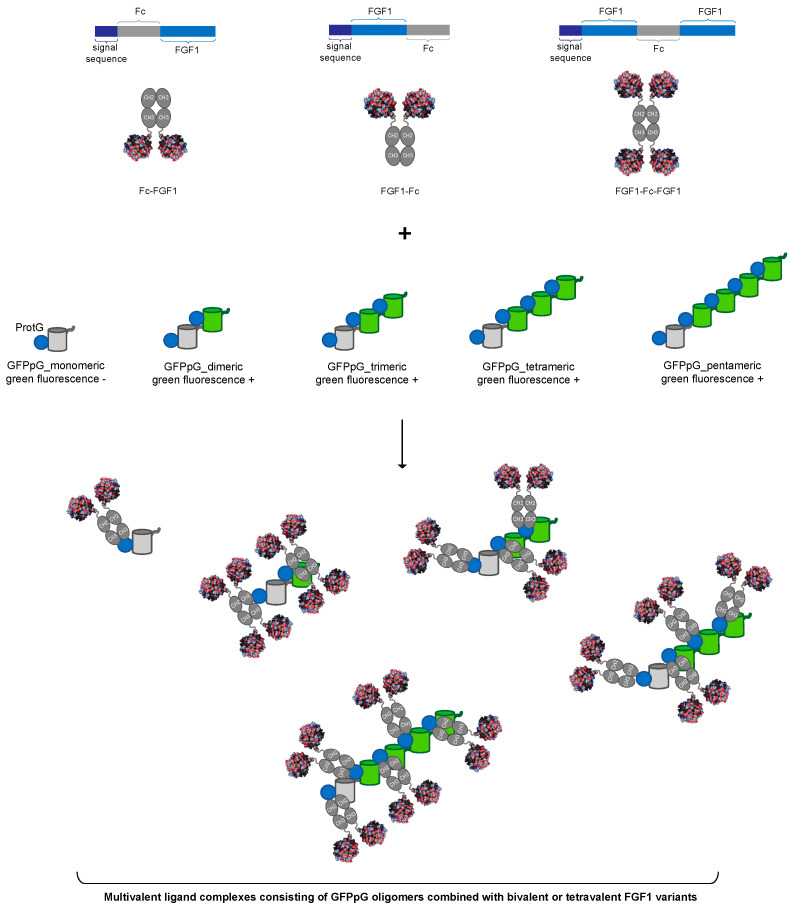
The strategy to generate multivalent ligand complexes consisting of bi- or tetravalent FGF1 variants and various GFPpolygonsProtG oligomers (GFPpG). In this approach, the monovalent FGF1 ligand is dimerized by N- or C-terminal fusion with the Fc fragment of human IgG1, yielding FGF1-Fc and Fc-FGF1. For development of the tetravalent FGF1 (FGF1-Fc-FGF1), a monovalent ligand was fused to both termini of the Fc fragment. GFPpolygons fused with protein G (GFPpG) constitute oligomerization scaffolds and, due to their fusion with protein G, ensure efficient Fc fragment binding. Mixing Fc-fused ligands and oligomeric GFPpG proteins leads to the assembly of multivalent FGF1 complexes.

To generate multivalent ligand complexes targeting FGFR1, we decided to exploit the stable interaction between the Fc fragment of human IgG1 and protein G. We employed FGF1, a natural monomeric FGFR1 ligand, as the FGFR1 recognizing molecule. We designed genetic constructs to produce bivalent FGF1 in two different orientations by fusing FGF1 at the N- or C-terminus to the Fc, yielding Fc-FGF1 and FGF1-Fc, respectively (Figure 1). To obtain an additional increase in the valency of FGF1, we developed construct allowing the production of tetravalent FGF1 (FGF1-Fc-FGF1) by attaching FGF1 to both termini of the Fc (Figure 1). An N-terminal signal peptide was incorporated to secrete the engineered recombinant proteins into the culture media.

As a scaffold for controlled oligomerization of distinct FGF1 fusions with the Fc, we employed GFPpolygons developed by Kim et al. [24]. GFPpolygons are GFP variants in which a single β-strand was transferred to the other part of the β-barrel. This results in an incomplete, non-fluorogenic β-barrel for monomeric GFPpolygons. However, intermolecular association between GFPpolygons provides the missing β-strand to yield fluorescent GFPpolygons oligomers (Figure 1) [24]. By fusing protein G to GFPpolygons (GFPpG) Kim et al. provided a universal platform for multivalent self-assembly of proteins bearing Fc fragments, including fusions of FGF1 with the Fc we developed (Figure 1). Using different GFPpG oligomeric states in a combination with bivalent Fc-linked FGF1, multivalent ligand complexes can be self-assembled.

### 3.2. Preparation of FGF1 Fusions with the Fc

**Figure 2:**
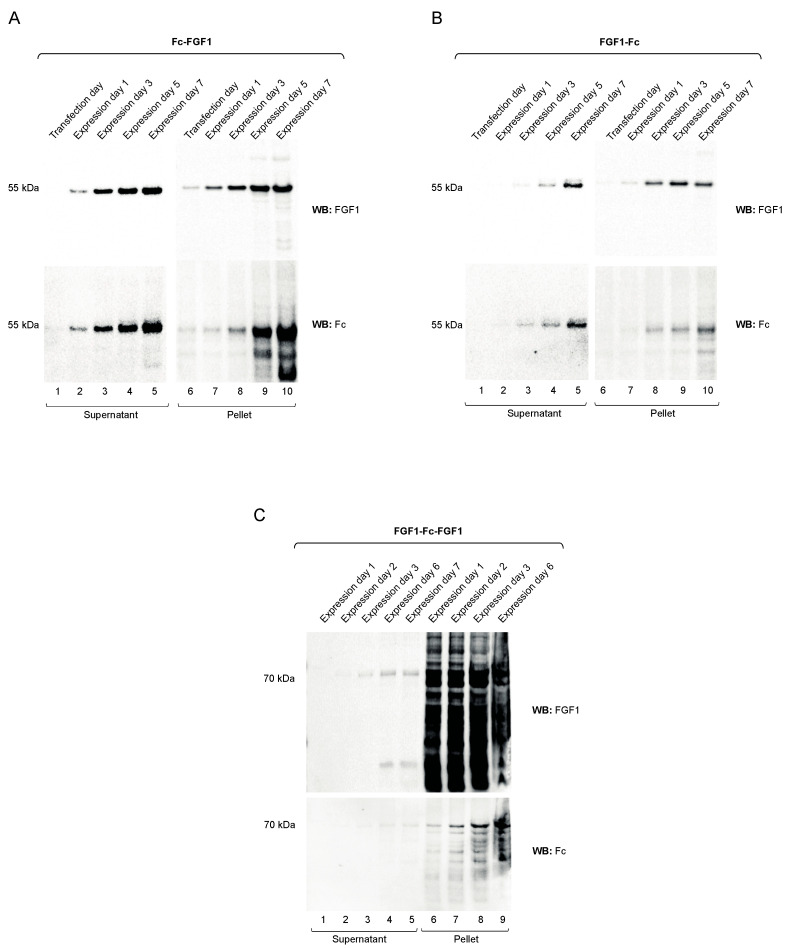
Expression of FGF1-fused to the Fc. Recombinant proteins: Fc-FGF1 (**A**), FGF1-Fc (**B**) and FGF1-Fc-FGF1 (**C**) were expressed in CHO-S cells. Levels of FGF1 variants at different stages of protein expression was monitored by Western blotting with antibodies recognizing FGF1 and Fc fragment.

FGF1-Fc, Fc-FGF1 and FGF1-Fc-FGF1 were expressed in Chinese hamster ovary (CHO) cells, allowing for the glycosylation of the Fc fragment and secretion of recombinant proteins into the culture medium, using protocols established in our lab for production of engineered anti-FGFR1 antibody fragments [19]. We monitored the production of FGF1 variants and their secretion into the culture medium using Western blotting with antibodies recognizing FGF1 and the Fc. As shown in Figure 2A,B (lanes 1–5), an efficient production and secretion of Fc-FGF1 and FGF1-Fc into the culture medium was observed. We also observed significant amounts of both Fc-FGF1 and FGF1-Fc in the cellular pellet, indicating that the release of these recombinant proteins by CHO cells was partially compromised (Figure 2A,B, lanes 6–10). FGF1-Fc-FGF1 was produced and secreted by CHO cells, but the expression level and the secretion efficiency of this variant were much lower than for the other recombinant proteins tested (Figure 2C).

**Figure 3:**
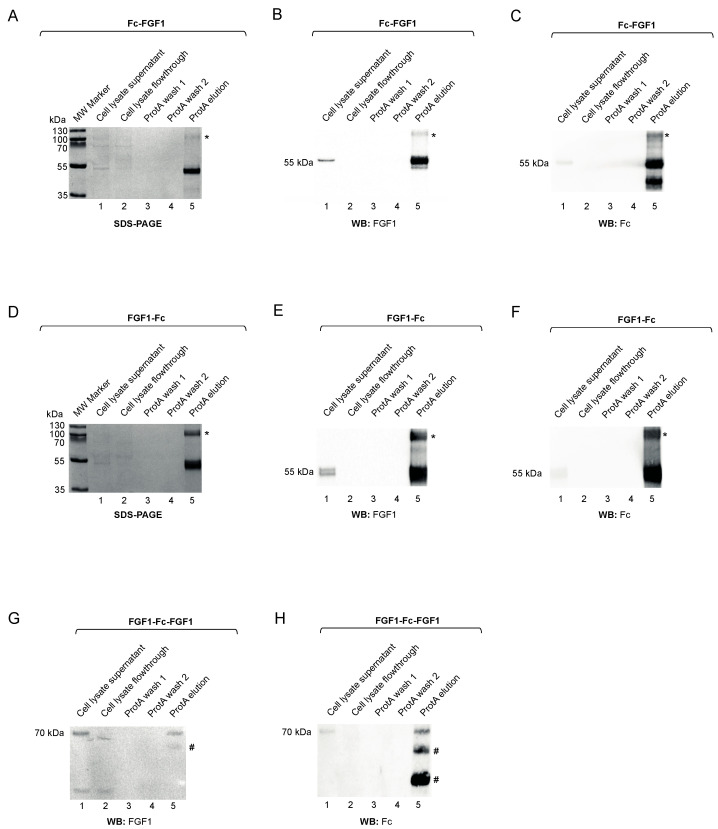
Purification of Fc-fused proteins. Fc-FGF1 (**A**), FGF1-Fc (**D**), and FGF1-Fc-FGF1 (**G**) were purified by protein A affinity chromatography. Protein purity was analyzed by SDS–PAGE (CBB staining) (**A**,**D**) and Western blotting with antibodies recognizing the FGF1 and Fc fragment (**B**,**C**,**E**–**H**). *—putative determination dimeric form of the protein, #—putative degradation products.

We employed affinity chromatography with Protein A beads to purify distinct Fc fusions of FGF1 from CHO culture supernatants. SDS-PAGE analyses revealed the successful purification of Fc-FGF1 and FGF1-Fc (Figure 3A,D, lane 5). We confirmed the identity of the purified proteins using Western blotting with antibodies specific for FGF1 (Figure 3B,E) and the Fc (Figure 3C,F). For both proteins, an additional band of higher MW, which was partially undissociated during the electrophoresis, corresponding to the Fc-FGF1 and FGF1-Fc dimer, was observed on SDS-PAGE and Western blots (Figure 3A–F, lane 5). The final efficiency of Fc-FGF1 and FGF1-Fc production was modest, as we obtained 0.728 and 0.45 mg of purified proteins from 1 L of CHO culture, respectively. The efficacy of FGF1-Fc-FGF1 purification was very low and we detected few degradation products (Figure 3G,H, lane 5).

In summary, highly purified bivalent Fc-FGF1 and FGF1-Fc variants were obtained. Since the purification efficiency of the tetravalent FGF1-Fc-FGF1 was very low, we decided to continue the study only with bivalent variants.

### 3.3. Biological Activity of FGF1 Fusions with the Fc Fragment

**Figure 4:**
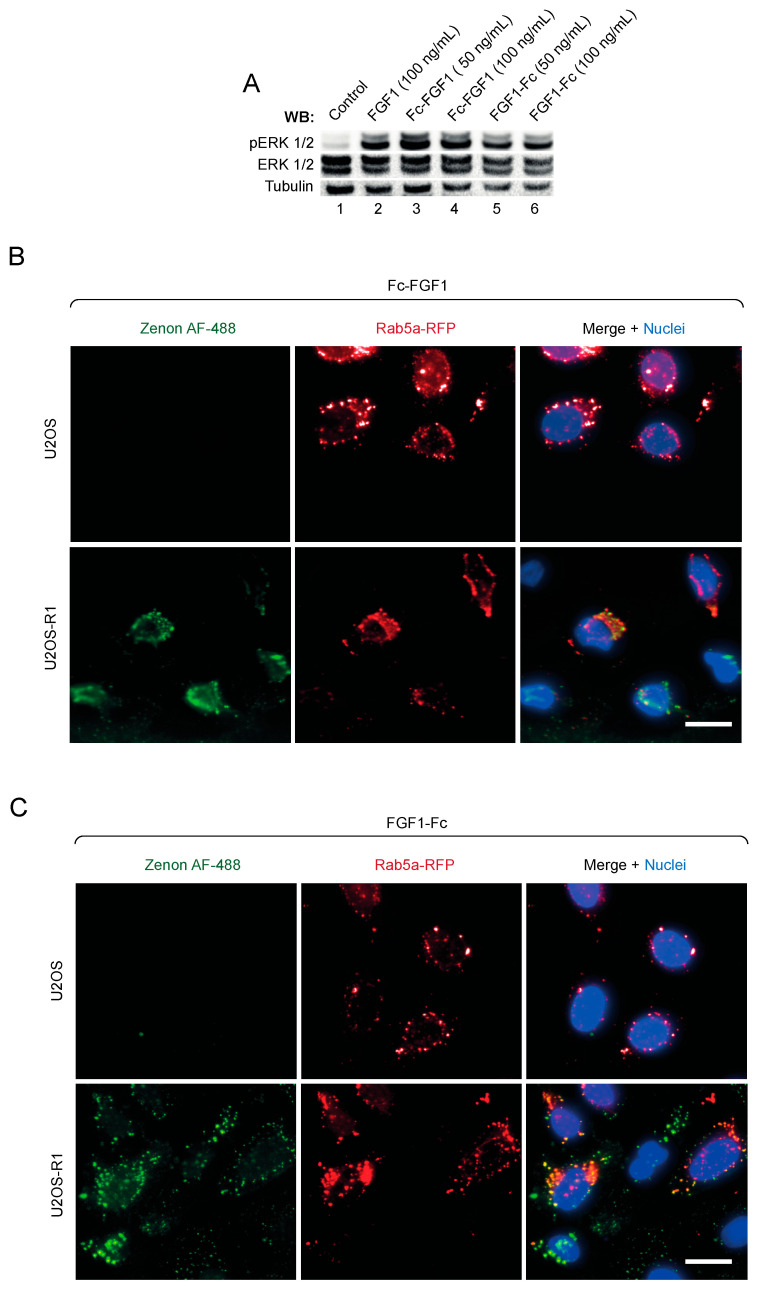
Biological activity of FGF1 fused to Fc fragment. (**A**) Serum-starved NIH3T3 cells were incubated for 15 min with FGF1, as a control, Fc-FGF1 or FGF1-Fc. Proteins were added in the presence of heparin (10 U/mL). Cells were lysed and the activation of receptor downstream ERK1/2 signaling was assessed with Western blotting (with antibodies recognizing phosphorylated ERK1/2 (pERK1/2)). The level of tubulin served as a loading control. (**B**,**C**) FGFR1-mediated internalization of FGF1 variants. U2OS (control) and U2OS-R1 cells were incubated with Fc-FGF1 (**B**) or FGF1-Fc (**C**) for 30 min at 37 °C. Nuclei were stained with NucBlue Live, and early endosomes were labeled by cell light early endosomes-RFP. Cells were fixed, and internalized recombinant proteins were visualized with Zenon AF-488 using the wide-field fluorescence microscope. The scale bar represents 20 μm.

For the biological relevance of assembled multivalent ligands, it is essential that the engineered FGF1 variants retain the receptor binding capacity. Therefore, we studied the ability of Fc-FGF1 and FGF1-Fc to bind FGFR1 and activate receptor-downstream signaling pathways. We employed serum-starved NIH3T3 fibroblasts that were treated with the wild type FGF1 or with Fc-FGF1 and FGF1-Fc. As shown in Figure 4A both Fc-FGF1 and FGF1-Fc activated FGFR1-downstream kinase ERK1/2 to a similar extent as the wild type FGF1.

Ligand-induced FGFR dimerization initiates the endocytosis of receptor-ligand complexes, typically via clathrin-mediated endocytosis [5,25,26,27]. We employed fluorescence microscopy to investigate whether FGF1-Fc and Fc-FGF1 are internalized upon incubation with U2OS-R1 cells, a model cell line stably producing FGFR1. Cells were initially transfected with a plasmid encoding Rab5a-RFP to fluorescently label early endosomes and subsequently incubated with non-labelled Fc-FGF1 or FGF1-Fc for 30 min. Cells were fixed and intracellular Fc-FGF1 and FGF1-Fc were detected with Zenon-AF488, a fluorescently labelled Fab fragment specifically recognizing the Fc. As shown in Figure 4B,C extensive colocalization of Zenon-AF488 and Rab5a-RFP signals was detected for both Fc-FGF1 and FGF1-Fc, indicating efficient endocytosis of the recombinant proteins tested. No internalization of both proteins studied was observed in control U2OS cells, characterized by undetectable level of FGFRs (Figure 4B,C).

These data demonstrate that Fc-FGF1 and FGF1-Fc are biologically active as they are able to bind FGFR, activate and induce receptor endocytosis.

### 3.4. Assembly of Multivalent Complexes of Engineered FGF1 and GFPpolygons

**Figure 5:**
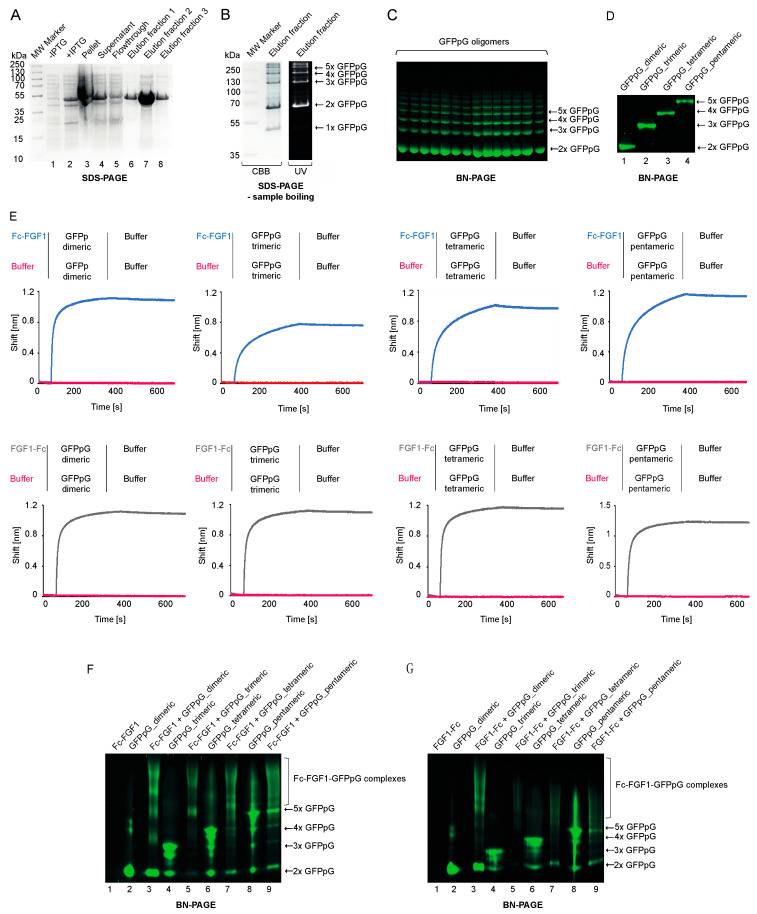
Preparation of GFPpG oligomers. (**A**–**C**). The mixture GFPpG oligomers was purified by nickel affinity chromatography and analyzed using SDS-PAGE (CBB staining). (**A**) Non-boiled GFPpG preparation were analyzed by SDS PAGE and visualized with CBB/fluorescence. (**B**) The assembly of GFPpG oligomers was assessed by BN-PAGE under UV light. (**C**,**D**) Purity of isolated GFPpG oligomers monitored with BN-PAGE. (**E**) Evaluation of Fc-FGF1 and FGF1-Fc interaction with GFPpG oligomers with BLI. FGF1 variants were chemically immobilized on BLI sensors and incubated with each GFPpG oligomer. Association and dissociation profiles were measured. (**F**,**G**) Complex formation between FGF1 fusions with the Fc and GFPpG oligomers. Recombinant proteins: Fc-FGF1 and FGFG1-Fc and various GFPpG variants (from dimer to pentamer) were mixed and incubated for 5–10 min at RT. Then, proteins mixture was subjected to BN-PAGE analysis under UV light.

In the next step we produced His-Tagged GFPpG in a bacterial expression system. By applying a low temperature during induction of protein production (16 °C), we observed more efficient assembly of higher oligomeric forms of GFPpG relative to higher growth temperatures (data not shown). We initially purified mixture of different oligomeric forms of GFPpG using nickel-charged resin (Figure 5A).

In agreement with Kim et al. [24], we observed that, in contrast to fully assembled β-barrels in GFPpG oligomers, monomeric GFPpG species are non-fluorescent (Figure 5B). Then, we applied Blue Native PAGE (BN-PAGE) to separate the distinct GFPpG. We excised particular oligomeric forms from the native gel (Figure 5C) and transferred the protein from the gel to the solution. Using this approach, we obtained milligram amounts of highly pure distinct oligomeric variants (from dimer to pentamer) of GFPpG (Figure 5D).

To assess the interaction between Fc-FGF1, FGF1-Fc and GFPpG, we used biolayer interferometry (BLI). Engineered FGF1 variants were immobilized on BLI sensors and incubated with distinct oligomeric forms of GFPpG. In all studied combinations an efficient complex assembly was detected (Figure 5E). The binding curves revealed very low dissociation rates, indicating the formation of stable complexes between Fc-FGF1, FGF1-Fc and different GFPpG variants.

To further confirm the assembly of multivalent complexes between Fc-FGF1, FGF1-Fc and distinct GFPpG oligomers, we employed BN-PAGE. To this end, FGF1 variants were mixed with particular oligomeric forms of GFPpG and the changes in GFPpG migration upon binding of Fc-FGF1 or FGF1-Fc were monitored. As shown in Figure 5F,G, efficient assembly of high molecular weight complexes between both Fc-FGF1, FGF1-Fc and different GFPpG oligomeric forms was detected.

All these data indicate the successful assembly of multivalent complexes targeting FGFR1, composed of FGF1 fused to the Fc fragment as a receptor recognizing molecule and GFPpG as an oligomerization scaffold. In the presented approach, complexes with different FGF1 orientation (due to diverse arrangement of FGF1 with the Fc fragment) and number of potential FGFR binding sites (due to distinct GFPpG forms) were obtained.

### 3.5. Biological Activity of Multivalent Complexes of Engineered FGF1 and GFPpolygons

**Figure 6:**
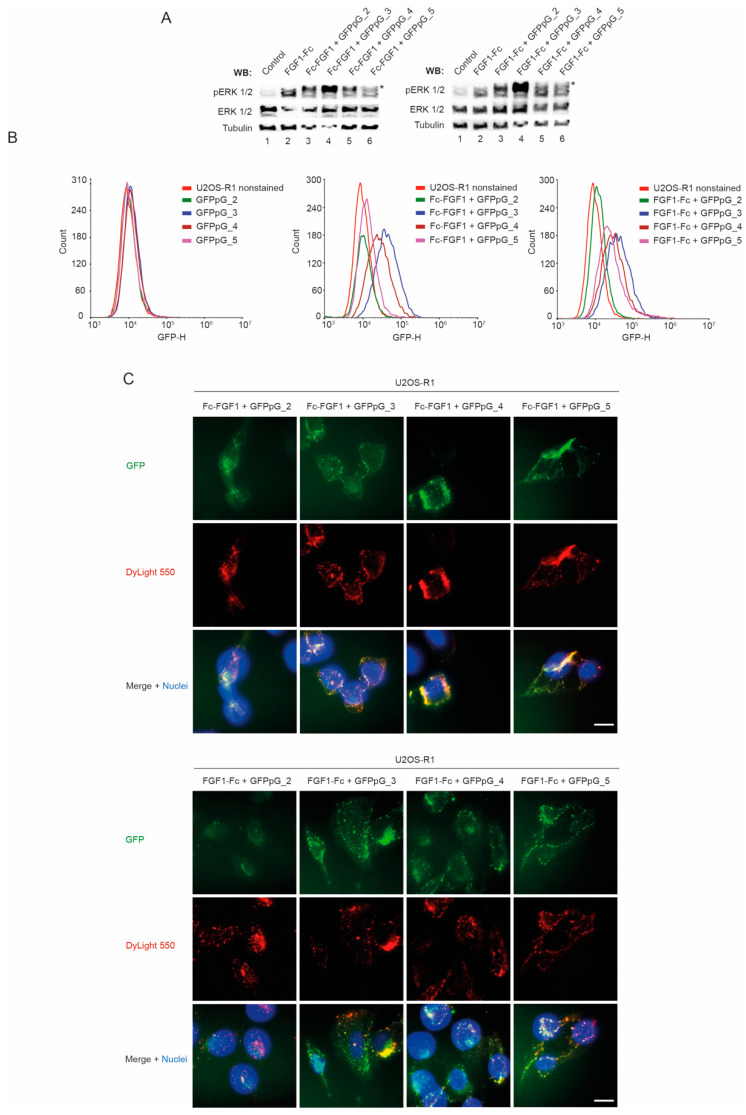
Biological activity of FGF1 and GFPpG complexes. (**A**) Serum-starved NIH3T3 cells were incubated for 15 min with Fc-FGF1 or FGF1-Fc and each of GFPpG variant. Mix of proteins were added in the presence of heparin (10 U/mL). Cells were lysed and activation of receptor-downstream ERK1/2 signaling was assessed with Western blotting (with antibodies recognizing phosphorylated ERK1/2 (pERK1/2)). The level of tubulin served as a loading control. (**B**) Efficiency of FGF1 and GFPpG complexes internalization analyzed by flow cytometry. Serum-starved U2OS-R1 cells were treated with fluorescent GFPpG variants and Fc-FGF1 or FGF1-Fc. After 20 min of incubation on ice, cells were transferred to 37 °C for 30 min, and then subsequently analyzed by NovoCyte 2060R Flow Cytometer. (**C**) FGFR1-mediated internalization of FGF1 and GFPpG multivalent complexes. U2OS-R1 cells were incubated with each of GFPpG variant (green) and DyLight 550 NHS Ester labeled Fc-FGF1 or FGF1-Fc (red) for 20 min on ice, and then for 45 min at 37 °C. Nuclei were stained with NucBlue Live. Cells were fixed and visualized using wide-field fluorescence microscope. The scale bar represents 20 μm. *—nonspecific signal from GFPpG.

Subsequently, we studied if multivalent FGFR1-targeting complexes retained ability to bind and activate FGFR1. Serum starved NIH3T3 cells were incubated with distinct complexes and activation of FGFR1-dependent signaling pathways was assessed with Western blotting. As shown in Figure 6A all multivalent complexes caused activation of FGFR1-downstream kinase ERK1/2.

To study if FGF1-Fc and Fc-FGF1 can fulfill the function of a targeting molecule within the multivalent FGFR1-targeting complexes we employed flow cytometry. U2OS-R1 cells were incubated with distinct GFPpG variants alone, or with their complexes with FGF1-Fc and Fc-FGF1. While GFPpG were incapable of entering the cells, all multivalent complexes containing either FGF1-Fc or Fc-FGF1 were efficiently internalized into U2OS-R1 cells (Figure 6B).

Next, we employed fluorescence microscopy to follow the cellular localization of the extracellularly administered multivalent FGFR1-targetting complexes. Complexes consisting of the dimeric GFPpG were localized in punctate structures indicating endosomes/lysosomes, typical for internalized FGF1 (Figure 6C). Interestingly, the complexes composed of trimeric, tetrameric and pentameric GFPpG were localized, besides punctate structures to the cell surface (Figure 6C).

All these data demonstrate that developed multivalent FGFR-targeting complexes are biologically active. Furthermore, our data indicate that multivalency of FGFR-targeting complexes may promote clustering of the receptor on the cell surface, limiting its endocytosis.

## 4. Discussion

Although most of RTKs activate similar intracellular pathways, the elicited cellular responses largely differ between individual RTK members. This phenomenon can be attributed to discrete differences in the amplitude and duration of signals transmitted via distinct intracellular pathways [28,29]. A number of regulatory mechanisms shape RTK-dependent signals, determining fate of cells. Regulation of RTK distribution in the plasma membrane is one of strategies for fine-tuning RTK-dependent signaling [10]. We have previously reported that by using engineered oligomeric FGFR1 ligands it is possible to control activity, endocytosis and cellular level of the receptor [21,22]. Importantly, the architecture of the oligomeric ligands (binding site on the receptor, affinity, oligomeric state, oligomerization scaffold used) played an important role in determining the outcome of FGFR1 modulation [21,22]. Therefore, we have sought to develop novel strategies to generate multivalent FGFR1 ligands.

So far, we have been able to obtain FGFR1 ligands having up to five receptor binding sites [22]. In this report, we present an approach that allows the efficient assembly of FGFR1-targeting oligomers with custom-selected, higher number of receptor binding sites. In principle, using this strategy it is possible to generate ligands with very high number of receptor binding sites, as it can be modulated both by the oligomeric state of the scaffold (GFPpG) and the valency the of the ligand (in our case, bivalent FGF1 fusions with the Fc, tetravalent FGF1-Fc-FGF1, etc.). Kim et al. described the efficient isolation of GFPpG composed of over ten units. Although we also observed the assembly of higher oligomeric states of GFPpG, we were able to efficiently separate high quantities of oligomers up to the pentamer. A slight modification of the GFPpG expression and isolation protocol would likely allow to isolate these higher state oligomers, but it was beyond the scope of this work.

Dikov et al. produced functional Fc-FGF1 in E. coli cells, implicating the feasibility of the strategy undertaken to fuse FGF1 with Fc [30]. In order to enable glycosylation of the Fc fragment we decided to produce FGF1 fusions with the Fc in CHO cells. We prepared N- and C-terminal fusions of FGF1 with the Fc to allow the assembly of FGFR1-targeting oligomers of distinct architecture. We were able to produce in mammalian cells and purify biologically active Fc-FGF1 and FGF1-Fc. Interestingly, we observed that large fractions of Fc-FGF1 and FGF1-Fc were trapped inside CHO cells, limiting overall yield. Although the engineered expression constructs are equipped with a functional signal peptide directing Fc-FGF1 and FGF1-Fc to the secretory route, it is likely that the intrinsic ability of FGF1 to employ unconventional secretion mechanisms partially outcompetes the ER targeting [19]. Consequently, the incorporation of a large Fc tag into FGF1 may limit the efficiency of its unconventional secretion, resulting in cytosolic localization of part of recombinant proteins. Clearly, further studies are required to understand in detail the limited efficiency of Fc-FGF1 and FGF1-Fc secretion.

The combination of Fc-tagged FGF1 and GFPpG resulted in the self-assembly of FGF1 oligomers formed through the Fc-protG interaction. Depending on the applied GFPpG oligomeric form, FGF1 complexes consisting of up to ten growth factor molecules were obtained, demonstrating the feasibility of the selected strategy. BLI experiments revealed that FGF1-Fc, Fc-FGF1 and GFPpG interactions are characterized by low koff, implicating high stability of the formed FGF1 oligomers. Notably, all developed FGF1 oligomers retained their biological activity, as they were able to stimulate FGFR1-dependent signaling pathways. Importantly, multivalent FGFR-targeting complexes caused clustering of the receptor on the cell surface, affecting cellular trafficking of the receptor. Similar effect we have recently observed for multivalent galectin-3, an extracellular lectin that binds sugar chains of FGFR1 [31,32].

FGF/FGFR signaling system is kept under tight control to ensure balanced stream of signals allowing for proper execution of metabolic and developmental processes. Developed multivalent complexes presented in this study can be used for fine-tuning FGFR signaling to evoke desired cellular activity, which may be helpful in understanding fundamentals of FGF/FGFR biology. Furthermore, some of multivalent FGFR-targeting complexes may turn out to be useful in clinics, e.g., in wound healing. Certainly, more in-depth studies are required to test the applicability of multivalent FGFR-targeting complexes.

## Data Availability

Orginal data are available pon request from corresponding author.
